# *In Vitro* Activity of Cefiderocol Against Meropenem-Nonsusceptible Gram-Negative Bacilli with Defined β-Lactamase Carriage: SIDERO-WT Surveillance Studies, 2014–2019

**DOI:** 10.1089/mdr.2022.0279

**Published:** 2023-07-31

**Authors:** Mark G. Wise, James A. Karlowsky, Meredith A. Hackel, Miki Takemura, Yoshinori Yamano, Roger Echols, Daniel F. Sahm

**Affiliations:** ^1^ IHMA, Schaumburg, Illinois, USA.; ^2^ Department of Medical Microbiology and Infectious Diseases, Max Rady College of Medicine, University of Manitoba, Winnipeg, Manitoba, Canada.; ^3^ Laboratory for Drug Discovery and Disease Research and Shionogi & Co., Ltd., Osaka, Japan.; ^4^ Research Planning Department, Shionogi & Co., Ltd., Osaka, Japan.; ^5^ Infectious Disease Drug Development Consulting, LLC, Easton, Connecticut, USA.

**Keywords:** cefiderocol, Gram-negative bacilli, CRE, carbapenem-resistant, Enterobacterales, *Pseudomonas aeruginosa*, *Acinetobacter baumannii*

## Abstract

We examined the *in vitro* susceptibility of meropenem-nonsusceptible Enterobacterales, *Pseudomonas aeruginosa*, and *Acinetobacter baumannii* complex isolates from five consecutive annual SIDERO-WT surveillance studies (2014–2019) to cefiderocol and comparator agents in the context of their carbapenemase carriage. 1,003 Enterobacterales, 1,758 *P. aeruginosa*, and 2,809 *A. baumannii* complex isolates from North America and Europe that were meropenem nonsusceptible (CLSI M100, 2022) were molecularly characterized for β-lactamase content by PCR followed by Sanger sequencing or by whole genome sequencing. Among Enterobacterales, 91.5% of metallo-β-lactamase (MBL)–producing, 98.4% of KPC-producing, 97.3% of OXA-48 group–producing, and 98.7% of carbapenemase-negative, meropenem-nonsusceptible isolates were cefiderocol susceptible (MIC ≤4 mg/L). Among *P. aeruginosa*, 100% of MBL-producing, 100% of GES carbapenemase-producing, and 99.8% of carbapenemase-negative, meropenem-nonsusceptible isolates were cefiderocol susceptible (MIC ≤4 mg/L). Among *A. baumannii* complex, 60.0% of MBL-producing, 95.6% of OXA-23 group-producing, 89.5% of OXA-24 group-producing, 100% of OXA-58 group-producing, and 95.5% of carbapenemase-negative, meropenem-nonsusceptible isolates were cefiderocol susceptible (MIC ≤4 mg/L). Cefiderocol was inactive against *A. baumannii* complex isolates carrying a PER or VEB β-lactamase (*n* = 103; 15.5% susceptible). Ceftazidime–avibactam and ceftolozane–tazobactam were inactive against MBL-carrying and *A. baumannii* complex isolates; ceftolozane–tazobactam was also inactive against serine carbapenemase–carrying Enterobacterales and *P. aeruginosa*. In summary, cefiderocol was highly active *in vitro* against Gram-negative isolates carrying MBLs and serine carbapenemases, as well as carbapenemase-negative, meropenem-nonsusceptible isolates.

## Introduction

Cefiderocol is a parenteral siderophore-cephalosporin conjugate whose current use is primarily as therapy for patients infected with carbapenem-resistant, multidrug-resistant (MDR), and difficult-to-treat resistant (DTR) Gram-negative bacilli when there are limited treatment options.^1–3^ In the United States, cefiderocol is approved for the treatment of adults with complicated urinary tract infections, including pyelonephritis, caused by susceptible Gram-negative bacilli (*Escherichia coli*, *Klebsiella pneumoniae*, *Proteus mirabilis*, *Enterobacter cloacae* complex, and *Pseudomonas aeruginosa*) for the treatment of hospital-acquired bacterial pneumonia and ventilator-associated bacterial pneumonia caused by Enterobacterales (*E. coli*, *K. pneumoniae*, *E. cloacae* complex, and *Serratia marcescens*), *P. aeruginosa*, and *Acinetobacter baumannii* complex.^4^ In Europe, cefiderocol is licensed for the treatment of infections due to aerobic Gram-negative organisms in adults with limited treatment options.^5^ As the prevalence of carbapenem-resistant, MDR, and DTR Gram-negative bacilli increases, and therapeutic options become increasingly limited, greater reliance will be placed on newer therapies such as cefiderocol to treat patients with recalcitrant infections.^6–10^

Previously we reported phenotypic *in vitro* susceptibility data from five consecutive annual surveillance studies (SIDERO-WT) that examined the activity of cefiderocol and comparator agents against 47,276 Gram-negative bacilli collected from clinical laboratories in North America and Europe from 2014 through 2019.^11^ That study showed that cefiderocol was highly active against antimicrobial nonsusceptible phenotypic subsets, including meropenem-nonsusceptible, ceftazidime-avibactam-nonsusceptible, and ceftolozane-tazobactam-nonsusceptible Enterobacterales and *P. aeruginosa*, as well as meropenem-nonsusceptible *A. baumannii* complex isolates. To further our understanding of cefiderocol's ability to overcome β-lactamase mediated resistance, we characterized the meropenem-nonsusceptible subsets of Enterobacterales, *P. aeruginosa*, and *A. baumannii* complex from the 2014 to 2019 SIDERO-WT surveillance studies for β-lactamase carriage.

## Materials and Methods

IRB approval and informed consent were not required because all isolates received into the study followed multiple subcultures and were completely de-identified. The secondary research use of de-identified isolates is considered exempt research according to the Regulations for the Protection of Human Subjects in Research of the U.S. Department of Health and Human Services, Office for Human Research Protections (45 CFR 46).

### Bacterial isolates

Five consecutive SIDERO-WT surveillance studies, sponsored by Shionogi & Co., Ltd., (Osaka, Japan), annually collected clinical isolates of Gram-negative bacilli in North America and Europe from November 2014 to December 2019 and included 31,896 Enterobacterales isolates, 7,700 *P. aeruginosa* isolates, 5,225 *A. baumannii* complex isolates, 2,030 *Stenotrophomonas maltophilia* isolates, and 425 *Burkholderia cepacia* complex isolates.^11^ IHMA (Schaumburg, IL) coordinated the studies, confirmed isolate identifications by MALDI-TOF mass spectrometry (Bruker Daltonics, Billerica, MA), and performed broth microdilution antimicrobial susceptibility testing of isolates.^12,13^

One-thousand and three meropenem-nonsusceptible Enterobacterales (meropenem MIC ≥2 mg/L), 1,758 meropenem-nonsusceptible *P. aeruginosa* (MIC ≥4 mg/L), and 2,809 meropenem-nonsusceptible *A. baumannii* complex (MIC ≥4 mg/L) isolates from the SIDERO-WT surveillance studies were subsequently subjected to molecular characterization, either by multiplex PCR followed by Sanger sequencing or by whole genome sequencing (WGS). Supplementary Table S1 summarizes the demographic data (specimen source, country of isolation, and year of isolation) associated with the meropenem-nonsusceptible Enterobacterales, *P. aeruginosa*, and *A. baumannii* complex isolates characterized in this study.

### Antimicrobial susceptibility testing

The CLSI broth microdilution method was used to determine isolate MICs,^12^ and CLSI MIC interpretative criteria were used to categorize MICs as susceptible, susceptible-dose dependent (cefepime tested against Enterobacterales), intermediate, or resistant.^13^ MIC interpretation by EUCAST^14^ and FDA^15^ criteria are included in Supplementary Tables S2–S4. Details of the antimicrobial susceptibility testing procedure were previously published.^11^

### β-Lactamase using PCR and sanger sequencing

Most meropenem-nonsusceptible Enterobacterales (*n* = 943), *P. aeruginosa* (*n* = 1,755), and *A. baumannii* complex (*n* = 2,726) isolates were interrogated by PCR for β-lactamase genes followed by Sanger sequencing. Isolate genomic DNA was obtained using the QIAamp^®^ DNA Mini protocol for the QiaCube (Qiagen, Gaithersburg, MD) following manufacturer's recommendations. Meropenem-nonsusceptible Enterobacterales, *P. aeruginosa*, and *A. baumannii* complex isolates were screened for the presence of *bla* encoding extended-spectrum β-lactamases (ESBLs) (TEM, SHV, CTX-M, including the five subtypes [CTX-M-1-type, CTX-M-2-type, CTX-M-8-type, CTX-M-9-type, and CTX-M-25-type], GES, VEB, and PER), AmpC β-lactamases (ACC, ACT, CMY, DHA, FOX, MIR, and MOX), MBLs (NDM, IMP, VIM, SPM, and GIM), Ambler class A serine carbapenemases (KPC and GES), and class D serine carbapenemases (OXA-23 group and OXA-58 group [*A. baumannii* complex only], OXA-24 group [*A. baumannii* complex and *P. aeruginosa*], and OXA-48 group [Enterobacterales and *A. baumannii* complex]) by multiplex PCR using published primers.^16–18^

Genes encoding the following enzymes were amplified with extragenic primers and sequenced: KPC, OXA-48-like, IMP, VIM, NDM, GES, VEB, PER, TEM, and SHV. *bla*_TEM_ and *bla*_SHV_ were first screened by limited sequencing to identify genes encoding enzymes containing amino acid substitutions common to TEM-type (amino acid positions 104, 164, 238, and 240) and SHV-type (amino acid positions 146, 179, 238, and 240) ESBLs. Only *bla*_SHV_ and *bla*_TEM_ that encoded ESBLs were completely sequenced.

For all β-lactamase genes sequenced, the deduced amino acid sequence was compared to available databases maintained by the NCBI (www.ncbi.nlm.nih.gov) to identify enzyme variants.

### Whole genome sequencing

One hundred and forty-six meropenem-nonsusceptible isolates that also tested with elevated cefiderocol MICs (≥4 mg/L [in 2018] and ≥8 mg/L [in 2019]), including 60 Enterobacterales, three *P. aeruginosa*, and 83 *A. baumannii* complex isolates, were subjected to WGS (in lieu of PCR and Sanger sequencing). Cells were pelleted from 3 mL liquid cultures grown overnight from one colony in Brain Heart Infusion broth (Sigma-Aldrich, St. Louis, MO) at 37°C with shaking. DNA was subsequently extracted using the DNeasy Ultraclean Microbial Extraction Kit (Qiagen). Sequencing libraries were prepared using the Illumina DNA Prep Library Preparation Kit (Illumina, San Diego, CA). Sequencing was performed on an Illumina HiSeq system using 2 × 150 bp paired-end reads with a target coverage depth of 100 times.

All analyses were carried out using the CLC Genomics Workbench, version 20 (Qiagen). For resistance gene identification, *de novo* assemblies of each genome were queried using the “find resistance” module, which interrogates the CGE database for resistance genes. β-lactamase genes with less than 100% sequence identity to a known nucleotide reference were translated to their deduced amino acid sequence and BLASTP searched against the RefSeq database in GenBank dedicated to β-lactamase nomenclature (BioProject 313047) to assign the enzyme variant.

## Results

Table 1 summarizes the identities and distribution of carbapenemases identified in the meropenem-nonsusceptible Enterobacterales, *P. aeruginosa*, and *A. baumannii* complex isolates stratified by geographic region (North America, Europe) and by year of collection (2014–2019). Among Enterobacterales, VIM was the most common MBL encountered (53.6% of MBL-positive isolates; 113/211), followed by NDM (45.5%; 96/211) and IMP (0.9%; 2/211). Geographically, MBL producers were more likely to be isolated in Europe, as 24.9% (198/794) of the meropenem-nonsusceptible Enterobacterales carried MBLs, compared to 6.2% (13/209) of North American isolates. In Europe, MBL-producing isolates were most numerous among those collected in Greece (*n* = 55), Russia (*n* = 38), Italy (*n* = 36), Turkey (*n* = 29), and Spain (*n* = 21).

**Table 1.** Carbapenemase Distribution by Geographic Region and Year Collected for the 1,003 Meropenem-Nonsusceptible Enterobacterales, 1,758 Meropenem-Nonsusceptible *Pseudomonas aeruginosa*, and 2,809 Meropenem-Nonsusceptible *Acinetobacter baumannii* Complex Isolates Molecularly Characterized in SIDERO-WT-2014-2019

Region/Year	Enterobacterales			P. aeruginosa			A. baumannii complex			
	MBL*^a^*	KPC*^b^*	OXA-48 group*^c^*	MBL*^d^*	KPC*^e^*	GES*^f^*	MBL*^g^*	OXA-23 group*^h^*	OXA-24 group*^i^*	OXA-58 group*^j^*
North America										
2014		5						37	21	
2015	1	14		1				33	19	
2016		31				1		83	39	1
2017		19		1		3		59	38	1
2018	3	26	2	1			1	47	26	3
2019	9	27	1	2		1	2	71	29	1
Total	13	122	3	5	0	5	3	330	172	6
Europe										
2014	19	32	2	11		2		218	47	10
2015	23	24	31	19			2	261	42	6
2016	39	66	82	65		4	3	318	74	45
2017	39	44	65	55		1		249	108	
2018	28	22	53	34		9	1	167	72	17
2019	50	77	38	38	16	13	16	299	74	23
Total	198	265	271	222	16	29	22	1,512	417	101
Grand total	211	387	274	227	16	34	25	1,842	589	107

^a^ Includes VIM (*n* = 113), NDM (*n* = 96), and IMP (*n* = 2); includes 18 isolates co-carrying OXA-48 group and 3 isolates co-carrying KPC.

^b^ Includes 4 isolates co-carrying VIM and 1 isolate co-carrying NDM.

^c^ Includes 15 isolates co-carrying NDM and 3 isolates co-carrying VIM.

^d^ Includes VIM (*n* = 200), IMP (*n* = 25), and NDM (*n* = 2); 16 isolates co-carried KPC and VIM.

^e^ Includes 16 isolates co-carrying VIM.

^f^ Only includes isolates harboring GES variants with reported carbapenemase activity (GES-2, −5, −15, and −20).

^g^ Includes NDM (*n* = 21), IMP (*n* = 2), and GIM (*n* = 2); includes 6 isolates co-carrying OXA-23 group and 4 isolates co-carrying OXA-58 group enzymes.

^h^ Includes 6 isolates co-carrying NDM, 19 co-carrying OXA-24 group, and 34 isolates co-carrying OXA-58 group enzymes.

^i^ Includes 19 isolates co-carrying OXA-23 group.

^j^ Includes 34 isolates co-carrying OXA-23 group.

MBL, metallo-β-lactamase.

KPC was identified in 387 Enterobacterales isolates, including four isolates co-carrying VIM and one isolate co-carrying NDM. Proportionally, KPC producers were more commonly identified in meropenem-nonsusceptible North American Enterobacterales (58.4%; 122/209) than in isolates from Europe (33.4%; 265/794).

OXA-48 group carbapenemases were identified in 274 Enterobacterales isolates, including 15 isolates co-carrying NDM and 3 isolates co-carrying VIM. Meropenem-nonsusceptible Enterobacterales from Europe were much more likely to carry OXA-48 group enzymes (34.1%; 271/794) than were isolates in North America (1.4%; 3/209).

Among meropenem-nonsusceptible *P. aeruginosa* isolates, 227 MBL carriers were detected, with VIM the most common (88.1%; 200/227), followed by IMP (11.0%; 25/227) and NDM (0.9%; 2/227). Sixteen VIM producers were observed that also carried KPC; all collected in 2019 in Europe (Russia, *n* = 15; Greece, *n* = 1). Overall, MBL–carrying *P. aeruginosa* were more prevalent in Europe, as 20.6% (222/1077) of the European meropenem-nonsusceptible isolates possessed an MBL compared to 0.7% (5/681) of North American isolates. In Europe, laboratories in Russia (*n* = 106) were the greatest contributors of MBL–carrying *P. aeruginosa*. In addition, 34 *P. aeruginosa* isolates in the collection were found to possess a GES enzyme variant with reported carbapenemase activity, including GES-2, GES-5, GES-15, and GES-20.

Twenty-five *A. baumannii* complex isolates were identified that carried MBLs; 21 isolates carried NDM (84.0%), two carried IMP (8.0%), and two carried GIM (German Imipenemase) (8.0%). *A. baumannii* complex isolates carrying OXA variants with carbapenemases activity were commonly identified in the collection. In total, 1,842 isolates were found harboring OXA-23 group enzymes (including 19 co-carrying OXA-24 group and 34 co-carrying OXA-58 group), 589 with OXA-24 group enzymes (including the 19 co-carrying OXA-23 group), and 107 carrying OXA-58 group enzymes (including the 34 co-carrying OXA-23 group). Of note, 99 *A. baumannii* complex isolates were PER-type ESBL positive; 54 of the 99 isolates co-carried an OXA-24 group enzyme, 40 isolates co-carried an OXA-23 group enzyme, and five isolates did not carry a carbapenemase. In addition, four *A. baumannii* isolates were identified harboring a VEB-type ESBL, each co-carrying an OXA-24 group enzyme.

Table 2 summarizes the *in vitro* activity of cefiderocol and comparator antimicrobial agents against 849 carbapenemase-producing and 154 carbapenemase-negative, meropenem-nonsusceptible Enterobacterales isolates. Cefiderocol inhibited 91.5% of MBL-producing isolates at a concentration (MIC) of ≤4 mg/L, the CLSI susceptible breakpoint for cefiderocol. A greater percentage of VIM carriers (96.5% susceptible; MIC_90_, 4 mg/L) than NDM carriers (85.4%; MIC_90_, 8 mg/L) were cefiderocol susceptible. All comparators for which CLSI currently publishes MIC breakpoints were inactive against MBL-producing isolates; 80.6% of isolates had colistin MICs ≤2 mg/L. The investigational β-lactam/β-lactamase inhibitor, aztreonam–avibactam (avibactam tested at a fixed concentration of 4 mg/L), was tested against the subset of meropenem-nonsusceptible Enterobacterales isolates collected in 2018 and 2019. This isolate subset included 92 MBL-producing isolates against which aztreonam–avibactam demonstrated potent *in vitro* activity (MIC_90_, 1 mg/L). Against both VIM- and NDM carriers, MIC_90_ values for all comparator agents except aztreonam–avibactam exceeded the highest agent concentration tested.

**Table 2.** Cumulative Antimicrobial Susceptibility Testing Results from SIDERO-WT Surveillance Study for Molecularly Characterized, Meropenem-Nonsusceptible Enterobacterales Isolates Collected in North America and Europe from 2014 to 2019

Genotype (no. of isolates)	Antimicrobial agent	MIC (mg/L)			CLSI MIC interpretation		
		Range	MIC_50_	MIC_90_	% Susceptible	% Intermediate	% Resistant
MBL (211)^a^	Cefiderocol	0.06–128	2	4	91.5	5.7	2.8
	Aztreonam–avibactam (*n* = 92)^e^	≤0.12–>8	0.25	1	NA^g^	NA	NA
	Cefepime^f^	0.25–>64	64	>64	3.8	2.8	93.4
	Ceftazidime–avibactam	0.5–>64	>64	>64	7.1	NA	92.9
	Ceftolozane–tazobactam	16–>64	>64	>64	0	0	100
	Ciprofloxacin	≤0.06–>8	>8	>8	5.7	5.2	89.1
	Colistin	≤0.12–>8	0.5	>8	NA	80.6	19.4
	Meropenem	2–>64	>16	>64	0	5.2	94.8
VIM (113)^a^	Cefiderocol	0.12–16	1	4	96.5	1.8	1.8
	Aztreonam–avibactam (*n* = 39)^e^	≤0.12–>8	≤0.12	2	NA	NA	NA
	Cefepime	0.25–>64	64	>64	6.2	4.4	89.4
	Ceftazidime–avibactam	1–>64	>64	>64	9.7	NA	90.3
	Ceftolozane–tazobactam	>16–>64	>64	>64	0	0	100
	Ciprofloxacin	≤0.06–>8	>8	>8	8.8	7.1	84.1
	Colistin	≤0.25–>8	0.5	>8	0	77.0	23.0
	Meropenem	2–>64	8	>64	0	8.0	92.0
NDM (96)^a^	Cefiderocol	0.12–128	2	8	85.4	10.4	4.2
	Aztreonam–avibactam (*n* = 51)^e^	≤0.12–>8	0.25	1	NA	NA	NA
	Cefepime	16–>64	>64	>64	0	0	100
	Ceftazidime–avibactam	0.5–>64	>64	>64	2.1	NA	97.9
	Ceftolozane–tazobactam	16–>64	>64	>64	0	0	100
	Ciprofloxacin	≤0.12–>8	>8	>8	1.0	3.1	95.8
	Colistin	≤0.12–>8	0.5	>8	NA	86.5	13.5
	Meropenem	2–>64	32	>64	0	2.1	97.9
KPC (382)^b^	Cefiderocol	0.015–8	1	4	98.4	1.6	0
	Aztreonam–avibactam (*n* = 150)^e^	≤0.12–>8	0.25	1	NA	NA	NA
	Cefepime	0.5–>64	64	>64	0.8	5.5	93.7
	Ceftazidime–avibactam	≤0.12–>64	1	4	97.6	NA	2.4
	Ceftolozane–tazobactam	2–>64	64	>64	0.3	1.0	98.7
	Ciprofloxacin	≤0.06–>8	>8	>8	2.4	1.8	95.8
	Colistin	≤0.12–>8	0.5	>8	0	70.2	29.8
	Meropenem	2–>64	>16	>64	0	3.1	96.9
OXA-48 group (256)^c^	Cefiderocol	0.015–16	0.5	4	97.3	2.3	0.4
	Aztreonam–avibactam (*n* = 87)^e^	≤0.12–>8	0.5	0.5	NA	NA	NA
	Cefepime	0.12–>64	>64	>64	14.1	7.8	78.1
	Ceftazidime–avibactam	0.12–>64	1	2	98.0	0	2.0
	Ceftolozane–tazobactam	0.25–>64	64	>64	11.7	4.3	84.0
	Ciprofloxacin	≤0.12–>8	>8	>8	5.1	1.6	93.4
	Colistin	≤0.12–>8	0.5	>8	0	75.0	25.0
	Meropenem	2–>64	16	64	0	18.0	82.0
Carbapenemase negative (154)^d^	Cefiderocol	0.008–8	0.5	2	98.7	1.3	0
	Aztreonam–avibactam (*n* = 50)^e^	≤0.12–>8	0.5	4	NA	NA	NA
	Cefepime	≤0.06–>64	16	>64	23.4	13.6	63.0
	Ceftazidime–avibactam	0.12–>64	2	32	84.4	0	15.6
	Ceftolozane–tazobactam	0.25–>64	16	>64	28.6	8.4	63.0
	Ciprofloxacin	≤0.06–>8	4	>8	28.6	5.2	66.2
	Colistin	≤0.12–>8	0.5	>8	0	71.4	28.6
	Meropenem	2–>64	8	>64	0	16.9	83.1

^a^ Isolates may also harbor serine carbapenemases, ESBLs, or AmpC-type enzymes.

^b^ Isolates may also harbor OXA-48-type carbapenemases, ESBLs, or AmpC-type enzymes but not MBLs.

^c^ Isolates may also harbor ESBLs or AmpC-type enzymes but not MBLs or KPC.

^d^ Isolates may harbor ESBLs or AmpC-type enzymes but not carbapenemases.

^e^ Aztreonam–avibactam was only tested in 2018 and 2019.

^f^ Percent cefepime susceptible-dose dependent values appear in the CLSI MIC interpretation, % Intermediate column.

^g^ NA, MIC breakpoints are not available.

ESBL, extended-spectrum β-lactamase.

Most KPC-producing Enterobacterales isolates (*n* = 382) were susceptible to cefiderocol (98.4%) and ceftazidime–avibactam (97.6%); the MIC_90_ for aztreonam–avibactam against the KPC-producing isolates tested (*n* = 150) was 1 mg/L. All other comparators for which CLSI currently publishes breakpoints were inactive against KPC-producing isolates; 70.2% of isolates had colistin MICs ≤2 mg/L. Similarly, most isolates harboring an OXA-48-like carbapenemase (*n* = 256) were susceptible to cefiderocol (97.3%) and ceftazidime–avibactam (98.0%); the MIC_90_ for aztreonam–avibactam against OXA-48-like–producing isolates (*n* = 87) was 0.5 mg/L. All other comparators for which CLSI currently publishes MIC breakpoints were inactive against OXA-48-like–producing isolates; 75.0% of isolates had colistin MICs ≤2 mg/L.

Among meropenem-nonsusceptible Enterobacterales, carbapenemase genes were not detected in 154 isolates (15.4%). Cefiderocol (98.7% susceptible; MIC_90_, 2 mg/L) inhibited 15% more carbapenemase-negative, meropenem-nonsusceptible Enterobacterales isolates than ceftazidime–avibactam (84.4% susceptible; MIC_90_, 32 mg/L). The MIC_90_ for aztreonam–avibactam against carbapenemase-negative, meropenem-nonsusceptible Enterobacterales isolates (*n* = 50) was 4 mg/L, two- to eight-fold (one to four doubling dilutions) higher than in carbapenemase-producing isolates. In comparison, the cefiderocol MIC_90_ was two- to four-fold (one to two doubling dilutions) lower (and the percent susceptible value marginally higher) for carbapenemase-negative, meropenem-nonsusceptible Enterobacterales than for carbapenemase-producing isolates.

Table 3 summarizes the *in vitro* activity of cefiderocol and comparator antimicrobial agents against 261 carbapenemase-producing and 1,497 carbapenemase-negative, meropenem-nonsusceptible *P. aeruginosa* isolates. Cefiderocol inhibited 100% of MBL-producing isolates at a concentration (MIC) of ≤4 mg/L, the CLSI susceptible breakpoint for cefiderocol. All comparators for which CLSI currently publishes MIC breakpoints were inactive against MBL-producing isolates; 98.7% of isolates had colistin MICs ≤2 mg/L. Aztreonam–avibactam was inactive (MIC_90_, >8 mg/L) against MBL–producing *P. aeruginosa* (*n* = 76). All *P. aeruginosa* isolates harboring a GES enzyme variant with reported carbapenemase activity (*n* = 34) were also cefiderocol susceptible (MIC_90_, 1 mg/L). This contrasts with the lower activities of ceftazidime–avibactam (76.5% susceptible) and aztreonam–avibactam (MIC_90_, 8 mg/L).

**Table 3.** Cumulative Antimicrobial Susceptibility Testing Results from SIDERO-WT Surveillance Study for Molecularly Characterized, Meropenem-Nonsusceptible *Pseudomonas aeruginosa* Isolates Collected in North America and Europe from 2014 to 2019

Genotype (no. of isolates)	Antimicrobial agent	MIC (mg/L)			CLSI MIC interpretation		
		Range	MIC_50_	MIC_90_	% Susceptible	% Intermediate	% Resistant
MBL (227)^a^	Cefiderocol	0.008–4	0.25	2	100	0	0
	Aztreonam–avibactam (*n* = 76)^d^	0.25–>8	>8	>8	NA^f^	NA	NA
	Cefepime	2–>64	32	>64	2.6	21.6	75.8
	Ceftazidime–avibactam	2–>64	32	>64	1.8	NA	98.2
	Ceftolozane–tazobactam	0.5–>64	>64	>64	0.9	0.0	99.1
	Ciprofloxacin	≤0.12–>8	>8	>8	1.8	2.2	96.0
	Colistin	≤0.25–4	1	2	0	98.7	1.3
	Meropenem	4–>64	64	>64	0	3.1	96.9
VIM (200)^a^	Cefiderocol	0.008–4	0.25	1	100	0	0
	Aztreonam–avibactam (*n* = 65)^d^	0.25–>8	>8	>8	NA	NA	NA
	Cefepime	2–>64	32	>64	3.0	24.5	72.5
	Ceftazidime–avibactam	2–>64	32	>64	2.0	NA	98.0
	Ceftolozane–tazobactam	0.5–>64	>64	>64	1.0	0	99.0
	Ciprofloxacin	≤0.12–>8	>8	>8	2.0	2.5	95.5
	Colistin	≤0.25–4	1	2	0	98.5	1.5
	Meropenem	4–>64	64	>64	0	3.5	96.5
IMP (25)^a^	Cefiderocol	0.12–4	2	4	100	0	0
	Aztreonam–avibactam (*n* = 9)^d^	8–>8	ND^e^	ND	NA	NA	NA
	Cefepime	>16–>64	>64	>64	0	0	100
	Ceftazidime–avibactam	>16–>64	>64	>64	0	NA	100
	Ceftolozane–tazobactam	>16–>64	>64	>64	0	0	100
	Ciprofloxacin	>8–>8	>8	>8	0	0	100
	Colistin	0.5–2	1	2	0	100	0
	Meropenem	8–>64	>64	>64	0	0	100
GES (carbapenemase) (34)^b^	Cefiderocol	0.06–1	0.25	1	100	0	0
	Aztreonam–avibactam (*n* = 23)^d^	2–8	4	8	NA	NA	NA
	Cefepime	4–>64	16	64	38.2	35.3	26.5
	Ceftazidime–avibactam	0.5–>64	4	64	76.5	NA	23.5
	Ceftolozane–tazobactam	0.5–>64	16	>64	11.8	32.4	55.9
	Ciprofloxacin	1–>8	8	>8	0	8.8	91.2
	Colistin	0.25–>8	1	1	0	97.1	2.9
	Meropenem	4–>64	>16	>64	0	8.8	91.2
Carbapenemase-negative (1,497)^c^	Cefiderocol	≤0.002–8	0.25	1	99.8	0.2	0
	Aztreonam–avibactam (*n* = 506)^d^	0.25–>8	8	>8	NA	NA	NA
	Cefepime	≤0.06–>64	8	32	56.3	22.4	21.3
	Ceftazidime–avibactam	≤0.06–>64	4	16	86.1	NA	13.9
	Ceftolozane–tazobactam	0.25–>64	1	8	89.0	3.1	7.9
	Ciprofloxacin	≤0.06–>8	2	>8	36.3	10.6	53.1
	Colistin	≤0.12–>8	1	1	0	98.5	1.5
	Meropenem	4–>64	8	>16	0	29.3	70.7

^a^ Isolates may also harbor serine carbapenemases, ESBLs, or AmpC-type enzymes.

^b^ Isolates may also harbor ESBLs or AmpC-type enzymes but not MBLs.

^c^ Isolates may harbor ESBLs or AmpC-type enzymes but not carbapenemases.

^d^ Aztreonam–avibactam was only tested in 2018 and 2019.

^e^ ND, not determined because isolates count was <10.

^f^ NA, MIC breakpoints are not available.

The majority of meropenem-nonsusceptible *P. aeruginosa* was carbapenemase negative (85.2%, 1,497/1,758). Cefiderocol was also highly active against meropenem-nonsusceptible, carbapenemase negative *P. aeruginosa* (99.8% susceptible; MIC_90_, 1 mg/L). Percent susceptible values for ceftolozane–tazobactam (89.0% susceptible) and ceftazidime–avibactam (86.1% susceptible) were both >10 percentage points lower than for cefiderocol against meropenem-nonsusceptible, carbapenemase-negative *P. aeruginosa*. Aztreonam–avibactam had an MIC_90_ value of >8 mg/L (*n* = 506) against meropenem-nonsusceptible, carbapenemase-negative *P. aeruginosa*.

Table 4 summarizes the *in vitro* activity of cefiderocol and comparator antimicrobial agents against 2,500 carbapenemase-producing and 309 carbapenemase-negative, meropenem-nonsusceptible *A. baumannii* complex isolates. Cefiderocol inhibited 60.0% of MBL-producing isolates (*n* = 25) at a concentration (MIC) of ≤4 mg/L, the CLSI susceptible breakpoint for cefiderocol; 96.0% of isolates had colistin MICs ≤2 mg/L. MIC interpretative criteria for isolates of *Acinetobacter* spp. have not been established for ceftazidime–avibactam (MIC_90_ >64 mg/L) and ceftolozane–tazobactam (MIC_90_ >64 mg/L).

**Table 4.** Cumulative Antimicrobial Susceptibility Testing Results from SIDERO-WT Surveillance Study for Molecularly Characterized, Meropenem-Nonsusceptible *Acinetobacter baumannii* Complex Isolates Collected in North America and Europe from 2014 to 2019

Genotype (no. of isolates)	Antimicrobial agent	MIC (mg/L)			CLSI MIC interpretation		
		Range	MIC_50_	MIC_90_	% Susceptible	% Intermediate	% Resistant
MBL (25)^a^	Cefiderocol	0.12–>64	4	8	60.0	32.0	8.0
	Ampicillin–sulbactam (*n* = 18)^e^	≤2–>64	>64	>64	5.6	11.1	83.3
	Cefepime (*n* = 7)^f^	>64–>64	>16	>64	0	0	100
	Ceftazidime–avibactam	>16–>64	>16	>64	NA^i^	NA	NA
	Ceftolozane–tazobactam (*n* = 7)^g^	>64–>64	ND^h^	ND	NA	NA	NA
	Ciprofloxacin	≤0.12–>8	>8	>8	28.0	0	72.0
	Colistin	≤0.25–8	0.5	0.5	0	96.0	4.0
	Meropenem	>16–>64	>16	>64	0	0	100
OXA-23 group (1,783)^b^	Cefiderocol	≤0.002–>256	0.25	2	95.6	1.9	2.6
	Ampicillin–sulbactam (*n* = 333)^e^	8–>64	32	64	6.0	27.3	66.7
	Cefepime (*n* = 1,449)^f^	0.12–>64	64	>64	1.7	9.0	89.3
	Ceftazidime–avibactam	1–>64	32	>64	NA	NA	NA
	Ceftolozane–tazobactam (*n* = 1,449)^g^	1–>64	32	>64	NA	NA	NA
	Ciprofloxacin	0.25–>8	>8	>8	0.1	0	99.9
	Colistin	≤0.25–>8	1	>8	0	81.4	18.6
	Meropenem	4–>64	64	>64	0	0.2	99.8
OXA-24 group (570)^b^	Cefiderocol	0.004–>256	0.5	8	89.5	1.9	8.6
	Ampicillin–sulbactam (*n* = 85)^e^	≤2–>64	16	64	27.1	27.1	45.9
	Cefepime (*n* = 485)^f^	1–>64	64	>64	8.2	24.5	67.2
	Ceftazidime–avibactam	4–>64	32	>64	NA	NA	NA
	Ceftolozane–tazobactam (*n* = 485)^g^	0.5–>64	16	>64	NA	NA	NA
	Ciprofloxacin	0.25–>8	>8	>8	0.7	0.0	99.3
	Colistin	≤0.25–>8	0.5	1	0	98.4	1.6
	Meropenem	4–>64	>64	>64	0	0.4	99.6
OXA-58 group (69)^b^	Cefiderocol	0.06–4	1	1	100	0	0
	Ampicillin–sulbactam (*n* = 9)^e^	8–>64	ND	ND	66.7	22.2	11.1
	Cefepime (*n* = 60)^f^	4–>64	32	64	16.7	28.3	55.0
	Ceftazidime–avibactam	8–>64	>64	>64	NA	NA	NA
	Ceftolozane–tazobactam (*n* = 60)^g^	1–>64	>64	>64	NA	NA	NA
	Ciprofloxacin	8–>8	>8	>8	0	0	100
	Colistin	≤0.25–>8	1	8	0	85.5	14.5
	Meropenem	4–>64	8	16	0	4.3	95.7
OXA-23 and 24 group (19)^b^	Cefiderocol	0.06–2	0.5	2	100	0	0
	Ampicillin–sulbactam (*n* = 18)^e^	8–>64	32	64	5.6	16.7	77.8
	Cefepime (*n* = 1)^f^	32	ND	ND	0	0	100
	Ceftazidime–avibactam	16–32	>16	>16	NA	NA	NA
	Ceftolozane–tazobactam (*n* = 1)^g^	16	ND	ND	NA	NA	NA
	Ciprofloxacin	>8–>8	>8	>8	0	0	100
	Colistin	≤0.25–>8	0.5	>8	0	89.5	10.5
	Meropenem	>16–>64	>16	>16	0	0	100
OXA-23 and 58 group (34)^b^	Cefiderocol	0.06–0.5	0.25	0.5	100	0	0
	Ampicillin–sulbactam (*n* = 13)^e^	16–64	32	64	0	15.4	84.6
	Cefepime (*n* = 21)^f^	16–>64	64	64	0	9.5	90.5
	Ceftazidime–avibactam	>16–>64	64	>64	NA	NA	NA
	Ceftolozane–tazobactam (*n* = 21)^g^	2–64	32	64	NA	NA	NA
	Ciprofloxacin	0.5–>8	>8	>8	2.9	0	97.1
	Colistin	≤0.25–>8	0.5	1	0	97.1	2.9
	Meropenem	4–>64	32	64	0	2.9	97.1
PER/VEB (103)^c^	Cefiderocol	0.12–>256	128	>256	15.5	10.7	73.8
	Ampicillin–sulbactam (*n* = 10)^e^	32–>64	64	>64	0	0	100
	Cefepime (*n* = 93)^f^	4–>64	>64	>64	1.1	0	98.9
	Ceftazidime–avibactam	2–>64	32	64	NA	NA	NA
	Ceftolozane–tazobactam (*n* = 93)^g^	16–>64	>64	>64	NA	NA	NA
	Ciprofloxacin	8–>8	>8	>8	0	0	100
	Colistin	≤0.25–2	1	1	0	100	0
	Meropenem	4–>64	64	>64	0	2.9	97.1
Carbapenemase-negative (309)^d^	Cefiderocol	0.008–>256	0.25	2	95.5	2.6	1.9
	Ampicillin–sulbactam (*n* = 62)^e^	≤2–>64	8	32	64.5	11.3	24.2
	Cefepime (*n* = 247)^f^	2–>64	16	>64	17.4	35.6	47.0
	Ceftazidime–avibactam	2–>64	32	>64	NA	NA	NA
	Ceftolozane–tazobactam (*n* = 247)^g^	≤0.06–>64	16	>64	NA	NA	NA
	Ciprofloxacin	≤0.12–>8	>8	>8	8.1	0.6	91.3
	Colistin	≤0.25–>8	0.5	1	0	98.1	1.9
	Meropenem	4–>64	16	64	0	19.4	80.6

^a^ Isolates may also harbor serine carbapenemases, ESBLs, or AmpC-type enzymes.

^b^ Isolates may also harbor ESBLs or AmpC-type enzymes but not MBL or other OXA carbapenemases than those noted.

^c^ Isolates may harbor OXA carbapenemases, ESBLs, or AmpC-type enzymes but not MBLs.

^d^ Isolates may harbor ESBLs or AmpC-type enzymes but not carbapenemases.

^e^ Ampicillin–sulbactam was only tested in 2019.

^f^ Cefepime data not available for 2019.

^g^ Ceftolozane–tazobactam was not tested in 2019.

^h^ ND, not determined because isolates count was <10.

^i^ NA, MIC breakpoints are not available.

Among meropenem-nonsusceptible *A. baumannii* complex isolates carrying OXA variants with carbapenemase activity, 1,783 isolates harbored OXA-23 group variants as their sole carbapenemase, 570 carried an OXA-24 group enzyme as their sole carbapenemase, and 69 carried an OXA-58 group enzyme as their sole carbapenemase. In addition, 19 isolates were identified that co-carried both OXA-23 and OXA-24 group and 34 isolates that co-carried both OXA-23 and OXA-58 group enzymes. Cefiderocol displayed potent *in vitro* activity against all these OXA groups with percent susceptible values ranging from 89.5% (OXA-24 group carriers) to 100% (OXA-58 group, OXA-23 and OXA-24 group co-carriers, OXA-23, and OXA-58 group co-carriers). Cefiderocol was also highly active against meropenem-nonsusceptible *A. baumannii* complex isolates for which a carbapenemase gene could not be detected (*n* = 309); 95.5% of these isolates were cefiderocol susceptible, >30 percentage points higher than the next most active comparator (ampicillin–sulbactam).

PER and VEB ESBLs are known to contribute to reduced cefiderocol susceptibility in *A. baumannii*.^19,20^ Ninety-nine *A. baumannii* complex isolates (3.5% of all meropenem nonsusceptible *A. baumannii* complex isolates tested) from the collection were determined to harbor PER; 54 of the 99 isolates co-carried an OXA-24 group enzyme, 40 isolates co-carried an OXA-23 group enzyme, and five isolates did not carry a carbapenemase. Four *A. baumannii* complex isolates were identified carrying VEB, each also carrying an OXA-24 group enzyme. Cefiderocol was largely inactive versus against the 103 PER/VEB-positive isolates with only 15.5% of isolates testing as susceptible (MIC_90_ ≥256 mg/L). The MIC_90_ values for all comparators against this isolate subset were greater than the highest concentration tested, except for ceftazidime–avibactam (MIC_90_ 64 mg/L) and colistin (MIC_90_ 1 mg/L).

## Discussion

Cefiderocol is noteworthy for its unique mechanism of Gram-negative bacterial cell entry. The chloro-catechol moiety of its C-3 side chain forms chelated complexes with ferric iron and promotes its transport across the outer membrane of Gram-negative bacilli using constitutive iron transport systems.^21^ Once in the periplasmic space, the iron dissociates and cefiderocol binds primarily to penicillin binding protein (PBP) 3, similar to other cephalosporins, and inhibits peptidoglycan synthesis.^21^ Cefiderocol is not hydrolyzed by most clinically important β-lactamases, including both serine β-lactamases (Ambler class A, class C, and class D) and MBLs, and is minimally affected by efflux-mediated resistance and porin deletions (two- to four-fold increases in cefiderocol MIC).^21–27^

We previously reported that the vast majority of Enterobacterales (99.8%), *P. aeruginosa* (99.9%), and *A. baumannii* complex (96.0%) collected across North America and Europe from 2014 to 2019 were susceptible to cefiderocol, including meropenem-nonsusceptible isolates.^11^ In addition, 91.6% isolates of ceftazidime-avibactam-nonsusceptible Enterobacterales and 97.7% of ceftolozane-tazobactam-nonsusceptible Enterobacterales were susceptible to cefiderocol, as were 100% of ceftazidime-avibactam-nonsusceptible and 99.8% of ceftolozane-tazobactam-nonsusceptible *P. aeruginosa.*^11^ Another international surveillance study collected clinical isolates from 66 hospitals in the United States and 18 European countries in 2020 and reported that 99.8% of 8,047 Enterobacterales, 98.2% of 169 carbapenem-resistant Enterobacterales, 89.2% of 37 ceftazidime-avibactam-resistant Enterobacterales, 99.6% of 2,282 *P. aeruginosa*, 97.3% of 256 XDR (extensively drug-resistant) *P. aeruginosa*, 91.6% of 83 ceftazidime-avibactam-resistant *P. aeruginosa*, 88.3% of 60 ceftolozane-tazobactam-resistant *P. aeruginosa*, 97.7% of 650 *Acinetobacter* species, and 95.8% of 306 meropenem-resistant *Acinetobacter* species isolates were cefiderocol susceptible.^28^

Cross-resistance between cefiderocol and other classes of antimicrobial agents has only rarely been reported.^29–31^ Isolates of Gram-negative bacilli resistant to other antimicrobial agents are generally susceptible to cefiderocol.^11,28^ Most isolates resistant to newer β-lactam/β-lactamase inhibitor combinations, including ceftazidime–avibactam, imipenem–relebactam, meropenem–vaborbactam, and ceftolozane–tazobactam, remain susceptible to cefiderocol.^11,28^ The newer β-lactam/β-lactamase inhibitor combinations were developed specifically to treat infections arising from serine carbapenemase–producing (KPC, OXA-48) Gram-negative bacilli. Unlike cefiderocol, none of the newer β-lactam/β-lactamase inhibitor combinations has activity against MBL–producing Gram-negative bacilli or carbapenem–resistant *A. baumannii* complex.^11,28^

Among MBL-producing isolates in this study, a greater percentage of VIM carriers (96.5%; MIC_90_, 4 mg/L) than NDM carriers (85.4%; MIC_90_, 8 mg/L) were cefiderocol susceptible. A similar observation has been reported by others.^10,23,25,27,32^ However, NDM production alone is likely not sufficient to cause cefiderocol resistance as elevated cefiderocol MICs in NDM producers were observed even in the presence of dipicolinic acid, an MBL inhibitor, and MICs were substantially reduced only when dipicolinic acid was used in combination with avibactam.^32^ Many isolates of NDM-producing Enterobacterales demonstrate cefiderocol MICs of ≤4 mg/L,^10,23,25,32^ and infections caused by NDM-producing Enterobacterales have been successfully treated with cefiderocol.^33^ In some isolates, simultaneous production of MBLs (NDM) and serine β-lactamases may lead to cefiderocol resistance that can be reversed *in vitro* by the addition of β-lactamase inhibitors, suggesting that resistance is likely mediated by combination of resistance mechanisms.^10^

In the current study, meropenem–nonsusceptible *P. aeruginosa* were highly susceptible to cefiderocol; 100% of MBL-producing, 100% of GES carbapenemase-producing, and 99.8% of carbapenemase-negative isolates were cefiderocol susceptible. Although it is unsurprising that newer approved β-lactam/β-lactamase-inhibitor combinations like ceftazidime–avibactam and ceftolozane–tazobactam were not active against MBL-carrying *P. aeruginosa*, the MIC_50_ value of >8 mg/L observed for aztreonam–avibactam suggests that it would also not be an effective therapeutic agent for this pathogen.

Among meropenem–nonsusceptible *A. baumannii* complex isolates, 60.0% of MBL-producing, 95.6% of OXA-23 group-producing, 89.5% of OXA-24 group-producing, 100% of OXA-58 group-producing, and 95.5% of carbapenemase-negative isolates were cefiderocol susceptible. Our report confirms previously published results.^10^ The β-lactamase inhibitor sulbactam has been shown to potentiate β-lactam antimicrobials against *A. baumannii*.^34^ Although ampicillin–sulbactam was tested on a limited number of organisms in this study solely from 2019 (*n* = 538), it exhibited poor *in vitro* activity against these meropenem nonsusceptible *A. baumannii* complex isolates as only 16.9% of this population was susceptible (MIC_90_, 64 mg/L).

PER and VEB ESBLs have been reported to contribute to reduced susceptibility in *A. baumannii*^10,19,20^; in the current study, 76 of 103 (73.8%) isolates of *A. baumannii* complex isolates that harbored PER or VEB were cefiderocol resistant and 11 (10.7%) were cefiderocol intermediate (cefiderocol MIC_90_, ≥256 mg/L). PER and VEB ESBLs were not identified in MBL–producing *A. baumannii* complex. Cefiderocol resistance due to PER in *A. baumannii* complex can be reversed by avibactam.^10^ Of interest, the eight *P. aeruginosa* isolates carrying PER, as well as the five carrying VEB, were all susceptible to cefiderocol, as were the two *K. pneumoniae* isolates identified carrying VEB.

Our study has three important limitations. First, we only investigated β-lactamases and no other potential mechanisms of resistance in meropenem-nonsusceptible isolates. However, current data suggest that efflux pump hyperexpression and porin channel mutations/loss, that can be responsible for carbapenem resistance, do not significantly influence the *in vitro* activity of cefiderocol.^21^ Second, evaluation of the mechanism(s) potentially responsible for the few cefiderocol-resistant, meropenem-nonsusceptible isolates identified was beyond the scope of this report. Other investigators have reported a limited number of isolates with increased cefiderocol MICs due to mutations (expression/function) in outer membrane TonB-dependent siderophore receptors (CirA and Fiu in Enterobacterales; PiuA and PiuD in *P. aeruginosa*; PirA and PiuA in *A. baumannii*) and siderophore expression-related genes (FecIRA operon), TonB-dependent iron transporter system energy generation component mutants/loss (TonB-ExbB-ExbD), PBP mutations, AmpC mutations (R2 loop mutations also associated with cross-resistance to ceftazidime–avibactam and ceftolozane–tazobactam), KPC variants, SHV-12, OXA-427, and the development of heteroresistance (primarily in *A. baumannii* complex isolates) on monotherapy in carbapenem-resistant isolates.^2,31,35–38^ A thorough review of cefiderocol resistance mechanisms was recently published.^39^ Third, we did not collect patient treatment or outcome information and do not know whether cefiderocol is in use in the hospitals that submitted isolates to SIDERO-WT. Given that cefiderocol was approved in the United States in November 2019 and in Europe in September 2020, this is unlikely.

We conclude that, based upon *in vitro* susceptibility testing data, cefiderocol may benefit the treatment of patients infected with carbapenem-nonsusceptible isolates of Enterobacterales, *P. aeruginosa*, and *A. baumannii* complex. In the current study, cefiderocol was highly active against Gram-negative isolates carrying MBLs and serine carbapenemases. The newer β-lactam/β-lactamase inhibitor combinations were inactive against isolates carrying serine carbapenemases (ceftolozane–tazobactam only), MBLs (ceftazidime–avibactam and ceftolozane–tazobactam), and *A. baumannii* complex (ceftazidime–avibactam and ceftolozane–tazobactam). Cefiderocol was also more active than ceftazidime–avibactam and ceftolozane–tazobactam against carbapenemase-negative, meropenem-nonsusceptible isolates of Enterobacterales and *P. aeruginosa*. Given that cefiderocol was only approved in both the United States and European countries in 2020, continued global surveillance will be important as clinical use of cefiderocol increases.

## Supplementary Material

Supplemental data

Supplemental data
