# Supplementary material for: In Vitro Activity of Cefiderocol Against Meropenem-Nonsusceptible Gram-Negative Bacilli with Defined β-Lactamase Carriage: SIDERO-WT Surveillance Studies, 2014–2019
Source: Microb Drug Resist. 2023 Jul 31;29(8):360–70. doi: 10.1089/mdr.2022.0279 (PMC10387160; doi:10.1089/mdr.2022.0279)
Supplement: Supplemental data [file Suppl_TableS1.docx]

**Table S1** Demographic information associated with the 1,003 meropenem-nonsusceptible Enterobacterales, 1,758 meropenem-nonsusceptible *Pseudomonas aeruginosa*, and 2,809 meropenem-nonsusceptible *Acinetobacter baumannii* complex isolates that were molecularly characterized from the SIDERO-WT surveillance study in North America and Europe from 2014 to 2019

|  |  | Enterobacterales | |  | | *Pseudomonas aeruginosa* | | |  | | *Acinetobacter baumannii* complex | | |
| --- | --- | --- | --- | --- | --- | --- | --- | --- | --- | --- | --- | --- | --- |
| Parameter | Parameter subset | no. of isolates with parameter | % of all isolates with parameter (*n*) | |  | | no. of isolates with parameter | % of all isolates with parameter (*n*) | |  | | no. of isolates with parameter | % of all isolates with parameter (*n*) |
| Specimen source | |  |  | |  | |  |  | |  | |  |  |
|  | Intra-abdominal | 209 | 3.6% (5,761) | |  | | 160 | 19.2% (833) | |  | | 370 | 50.4% (734) |
|  | Urinary tract | 109 | 2.4% (4,510) | |  | | 101 | 16.4% (614) | |  | | 156 | 59.8% (261) |
|  | Lower respiratory tract | 304 | 3.8% (7,940) | |  | | 994 | 27.5% (3,621) | |  | | 1447 | 61.6% (2,348) |
|  | Skin and soft tissue | 152 | 3.3% (4,593) | |  | | 225 | 17.2% (1,305) | |  | | 501 | 47.1% (1,064) |
|  | Bloodstream | 208 | 2.5% (8,368) | |  | | 224 | 19.8% (1,129) | |  | | 262 | 38.2% (686) |
|  | Other | 21 | 2.9% (724) | |  | | 54 | 27.3% (198) | |  | | 73 | 55.3% (132) |
|  | Total | 1,003 | 3.1% (31,896) | |  | | 1,758 | 22.8% (7,700) | |  | | 2,809 | 53.8% (5,225) |
|  |  |  |  | |  | |  |  | |  | |  |  |
| Country of isolation | |  |  | |  | |  |  | |  | |  |  |
|  | Canada | 13 | 0.6% (2,038) | |  | | 87 | 18.9% (461) | |  | | 20 | 8.2% (243) |
|  | United States | 196 | 1.5% (12,842) | |  | | 594 | 19.2% (3,087) | |  | | 678 | 38.6% (1,756) |
|  |  |  |  | |  | |  |  | |  | |  |  |
|  | Czech Republic | 8 | 0.6% (1,350) | |  | | 100 | 29.9% (334) | |  | | 28 | 13.1% (213) |
|  | France | 4 | 0.2% (1,689) | |  | | 59 | 14.1% (419) | |  | | 40 | 13.8% (289) |
|  | Germany | 20 | 1.0% (1,918) | |  | | 110 | 23.3% (473) | |  | | 51 | 19.2% (265) |
|  | Greece | 173 | 11.6% (1,490) | |  | | 68 | 22.4% (304) | |  | | 407 | 96.9% (420) |
|  | Hungary | 13 | 1.2% (1,114) | |  | | 122 | 43.9% (278) | |  | | 151 | 71.9% (210) |
|  | Italy | 201 | 10.3% (1,947) | |  | | 118 | 25.5% (463) | |  | | 432 | 85.5% (505) |
|  | Russia | 149 | 8.6% (1,735) | |  | | 212 | 40.8% (520) | |  | | 347 | 79.4% (437) |
|  | Spain | 74 | 3.8% (1,930) | |  | | 123 | 25.5% (482) | |  | | 238 | 70.8% (336) |
|  | Sweden | 1 | 0.2% (581) | |  | | 10 | 8.1% (124) | |  | | 6 | 60.0% (10) |
|  | Turkey | 128 | 9.3% (1,376) | |  | | 103 | 27.9% (369) | |  | | 391 | 90.5% (432) |
|  | United Kingdom | 23 | 1.5% (1,565) | |  | | 52 | 13.5% (386) | |  | | 20 | 18.3% (109) |
|  | Total | 1,003 | 3.2% (31,575)^a^ | |  | | 1,758 | 22.8% (7,700) | |  | | 2,809 | 53.8 (5,225) |
|  |  |  |  | |  | |  |  | |  | |  |  |
| Year of isolation | |  |  | |  | |  |  | |  | |  |  |
|  | 2014 | 59 | 2.5% (2,394) | |  | | 94 | 20.0% (471) | |  | | 377 | 76.9% (490) |
|  | 2015 | 98 | 2.1% (4,644) | |  | | 267 | 24.5% (1,092) | |  | | 405 | 58.8% (689) |
|  | 2016 | 272 | 3.4% (8,015) | |  | | 452 | 24.2% (1,864) | |  | | 625 | 55.1% (1,135) |
|  | 2017 | 197 | 3.4% (5,802) | |  | | 345 | 22.4% (1,541) | |  | | 491 | 53.0% (927) |
|  | 2018 | 157 | 3.2% (4,948) | |  | | 257 | 20.6% (1,245) | |  | | 373 | 39.6% (943) |
|  | 2019 | 220 | 3.6% (6,093) | |  | | 343 | 23.1% (1,487) | |  | | 538 | 51.7% (1,041) |
|  | Total | 1,003 | 3.1% (31,896) | |  | | 1,758 | 22.8% (7,700) | |  | | 2,809 | 53.8 (5,225) |

^a^Meropenem-nonsusceptible Enterobacterales were not identified among isolates from Austria (*n*=35), Belgium (*n*=83), Croatia (*n*=3), Denmark (*n*=36), Latvia (*n*=3), Lithuania (*n*=5), Netherlands (*n*=45), Poland (*n*=12), Romania (*n*=11), Serbia (*n*=1), and Slovenia (*n*=1). One meropenem-nonsusceptible isolate from Portugal (*n*=86) was not characterized.
