# Supplementary material for: In Vitro Activity of Cefiderocol Against Meropenem-Nonsusceptible Gram-Negative Bacilli with Defined β-Lactamase Carriage: SIDERO-WT Surveillance Studies, 2014–2019
Source: Microb Drug Resist. 2023 Jul 31;29(8):360–70. doi: 10.1089/mdr.2022.0279 (PMC10387160; doi:10.1089/mdr.2022.0279)
Supplement: Supplemental data [file Suppl_TableS2-S4.docx]

**Table S2.** EUCAST and FDA susceptibility-testing interpretation for molecularly characterized, meropenem-nonsusceptible Enterobacterales isolates from SIDERO-WT surveillance study collected in North America and Europe from 2014 to 2019

| Genotype (no. of isolates) | Antimicrobial agent | EUCAST MIC interpretation | | |  | FDA MIC interpretation | | |
| --- | --- | --- | --- | --- | --- | --- | --- | --- |
|  |  | % S | % I | % R |  | % S | % I | % R |
| MBL (211)^a^ | Cefiderocol | 68.2 | NA | 31.8 |  | 91.5 | 5.7 | 2.8 |
|  | Aztreonam-avibactam (*n*=92)^e^ | NA | NA | NA |  | NA^f^ | NA | NA |
|  | Cefepime | 2.4 | 1.4 | 96.2 |  | 3.8 | 2.8 | 93.4 |
|  | Ceftazidime-avibactam | 7.1 | NA | 92.9 |  | 7.1 | NA | 92.9 |
|  | Ceftolozane-tazobactam | 0 | NA | 100 |  | 0 | 0 | 100 |
|  | Ciprofloxacin | 5.7 | 5.2 | 89.1 |  | 5.7 | 5.2 | 89.1 |
|  | Colistin | 80.6 | NA | 19.4 |  | NA | NA | NA |
|  | Meropenem | 5.2 | 27.0 | 67.8 |  | 0 | 5.2 | 94.8 |
|  |  |  |  |  |  |  |  |  |
| VIM (113)^a^ | Cefiderocol | 80.5 | NA | 19.5 |  | 96.5 | 1.8 | 1.8 |
|  | Aztreonam-avibactam (*n*=39)^e^ | NA | NA | NA |  | NA | NA | NA |
|  | Cefepime | 4.4 | 1.8 | 93.8 |  | 6.2 | 4.4 | 89.4 |
|  | Ceftazidime-avibactam | 9.7 | NA | 90.3 |  | 9.7 | NA | 90.3 |
|  | Ceftolozane-tazobactam | 0 | NA | 100 |  | 0 | 0 | 100 |
|  | Ciprofloxacin | 8.8 | 7.1 | 84.1 |  | 8.8 | 7.1 | 84.1 |
|  | Colistin | 77.0 | NA | 23.0 |  | NA | NA | NA |
|  | Meropenem | 8.0 | 45.1 | 46.9 |  | 0 | 8.0 | 92.0 |
|  |  |  |  |  |  |  |  |  |
| NDM (96)^a^ | Cefiderocol | 53.1 | NA | 46.9 |  | 85.4 | 10.4 | 4.2 |
|  | Aztreonam-avibactam (*n*=51)^e^ | NA | NA | NA |  | NA | NA | NA |
|  | Cefepime | 0 | 0 | 100 |  | 0 | 0 | 100 |
|  | Ceftazidime-avibactam | 2.1 | NA | 97.9 |  | 2.1 | NA | 97.9 |
|  | Ceftolozane-tazobactam | 0 | NA | 100 |  | 0 | 0 | 100 |
|  | Ciprofloxacin | 1.0 | 3.1 | 95.8 |  | 1.0 | 3.1 | 95.8 |
|  | Colistin | 86.5 | NA | 13.5 |  | NA | NA | NA |
|  | Meropenem | 2.1 | 4.2 | 93.8 |  | 0 | 2.1 | 97.9 |
|  |  |  |  |  |  |  |  |  |
| KPC (382)^b^ | Cefiderocol | 80.9 | NA | 19.1 |  | 98.4 | 1.6 | 0 |
|  | Aztreonam-avibactam (*n*=150)^e^ | NA | NA | NA |  | NA | NA | NA |
|  | Cefepime | 0.3 | 2.1 | 97.6 |  | 0.8 | 5.5 | 93.7 |
|  | Ceftazidime-avibactam | 97.6 | NA | 2.4 |  | 97.6 | NA | 2.4 |
|  | Ceftolozane-tazobactam | 0.3 | NA | 99.7 |  | 0.3 | 1.0 | 98.7 |
|  | Ciprofloxacin | 2.4 | 1.8 | 95.8 |  | 2.4 | 1.8 | 95.8 |
|  | Colistin | 70.2 | NA | 29.8 |  | NA | NA | NA |
|  | Meropenem | 3.1 | 23.6 | 73.3 |  | 0 | 3.1 | 96.9 |
|  |  |  |  |  |  |  |  |  |
| OXA-48 (256)^c^ | Cefiderocol | 80.1 | NA | 19.9 |  | 97.3 | 2.3 | 0.4 |
|  | Aztreonam-avibactam (*n*=87)^e^ | NA | NA | NA |  | NA | NA | NA |
|  | Cefepime | 8.2 | 10.2 | 81.6 |  | 14.1 | 7.8 | 78.1 |
|  | Ceftazidime-avibactam | 98.0 | NA | 2.0 |  | 98.0 | 0 | 2.0 |
|  | Ceftolozane-tazobactam | 11.7 | NA | 88.3 |  | 11.7 | 4.3 | 84.0 |
|  | Ciprofloxacin | 5.1 | 1.6 | 93.4 |  | 5.1 | 1.6 | 93.4 |
|  | Colistin | 75.0 | NA | 25.0 |  | NA | NA | NA |
|  | Meropenem | 18.0 | 30.9 | 51.2 |  | 0 | 18.0 | 82.0 |
|  |  |  |  |  |  |  |  |  |
| Carbapenemase-negative (154)^d^ | Cefiderocol | 91.6 | NA | 8.4 |  | 98.7 | 1.3 | 0 |
|  | Aztreonam-avibactam (*n*=50)^e^ | NA | NA | NA |  | NA | NA | NA |
|  | Cefepime | 20.1 | 10.4 | 69.5 |  | 23.4 | 13.6 | 63.0 |
|  | Ceftazidime-avibactam | 84.4 | NA | 15.6 |  | 84.4 | 0 | 15.6 |
|  | Ceftolozane-tazobactam | 28.6 | NA | 71.4 |  | 28.6 | 8.4 | 63.0 |
|  | Ciprofloxacin | 28.6 | 5.2 | 66.2 |  | 28.6 | 5.2 | 66.2 |
|  | Colistin | 71.4 | NA | 28.6 |  | NA | NA | NA |
|  | Meropenem | 16.9 | 39.6 | 43.5 |  | 0 | 16.9 | 83.1 |

^a^Isolates may also harbor serine carbapenemases, ESBLs, or AmpC-type enzymes.

^b^Isolates may also harbor OXA-48-type carbapenemases, ESBLs or AmpC-type enzymes but not MBLs.

^c^Isolates may also harbor ESBLs or AmpC-type enzymes but not MBLs or KPC.

^d^Isolates may harbor ESBLs or AmpC-type enzymes but not carbapenemases.

^e^Aztreonam-avibactam was only tested in 2018 and 2019.

^f^NA, MIC breakpoints are not available.

**Table S3.** EUCAST and FDA susceptibility-testing interpretation for molecularly characterized, meropenem-nonsusceptible *Pseudomonas aeruginosa* isolates from SIDERO-WT surveillance study collected in North America and Europe from 2014 to 2019

| Genotype (no. of isolates) | Antimicrobial agent | EUCAST MIC interpretation | | |  | FDA MIC interpretation | | |
| --- | --- | --- | --- | --- | --- | --- | --- | --- |
|  |  | % S | % I | % R |  | % S | % I | % R |
| MBL (227)^a^ | Cefiderocol | 97.4 | NA | 2.6 |  | 86.8 | 10.6 | 2.6 |
|  | Aztreonam-avibactam (*n*=76)^d^ | NA^e^ | NA | NA |  | NA | NA | NA |
|  | Cefepime | NA | 2.6 | 97.4 |  | 2.6 | NA | 97.4 |
|  | Ceftazidime-avibactam | 1.8 | NA | 98.2 |  | 1.8 | NA | 98.2 |
|  | Ceftolozane-tazobactam | 0.9 | NA | 99.1 |  | 0.9 | 0 | 99.1 |
|  | Ciprofloxacin | NA | 1.8 | 98.2 |  | 1.8 | 2.2 | 96.0 |
|  | Colistin | 100 | NA | 0 |  | NA | NA | NA |
|  | Meropenem | 0 | 6.2 | 93.8 |  | 0 | 3.1 | 96.9 |
|  |  |  |  |  |  |  |  |  |
| VIM (200)^a^ | Cefiderocol | 99.0 | NA | 1.0 |  | 94.5 | 4.5 | 1.0 |
|  | Aztreonam-avibactam (*n*=65)^d^ | NA | NA | NA |  | NA | NA | NA |
|  | Cefepime | NA | 3.0 | 97.0 |  | 3.0 | NA | 97.0 |
|  | Ceftazidime-avibactam | 2.0 | NA | 98.0 |  | 2.0 | NA | 98.0 |
|  | Ceftolozane-tazobactam | 1.0 | NA | 99.0 |  | 1.0 | 0 | 99.0 |
|  | Ciprofloxacin | NA | 2.0 | 98.0 |  | 2.0 | 2.5 | 95.5 |
|  | Colistin | 100 | NA | 0 |  | NA | NA | NA |
|  | Meropenem | 0 | 5.5 | 94.5 |  | 0 | 3.5 | 96.5 |
|  |  |  |  |  |  |  |  |  |
| IMP (25)^a^ | Cefiderocol | 88.0 | NA | 12.0 |  | 32.0 | 56.0 | 12.0 |
|  | Aztreonam-avibactam (*n*=9)^d^ | NA | NA | NA |  | NA | NA | NA |
|  | Cefepime | NA | 0 | 100 |  | 0 | NA | 100 |
|  | Ceftazidime-avibactam | 0 | NA | 100 |  | 0 | NA | 100 |
|  | Ceftolozane-tazobactam | 0 | NA | 100 |  | 0 | 0 | 100 |
|  | Ciprofloxacin | NA | 0 | 100 |  | 0 | 0 | 100 |
|  | Colistin | 100 | NA | 0 |  | NA | NA | NA |
|  | Meropenem | 0 | 12.0 | 88.0 |  | 0 | 0 | 100 |
|  |  |  |  |  |  |  |  |  |
| GES (carbapenemase) (34)^b^ | Cefiderocol | 100 | NA | 0 |  | 100 | 0 | 0 |
|  | Aztreonam-avibactam (*n*=23)^d^ | NA | NA | NA |  | NA | NA | NA |
|  | Cefepime | NA | 38.2 | 61.8 |  | 38.2 | NA | 61.8 |
|  | Ceftazidime-avibactam | 76.5 | NA | 23.5 |  | 76.5 | NA | 23.5 |
|  | Ceftolozane-tazobactam | 11.8 | NA | 88.2 |  | 11.8 | 32.4 | 55.9 |
|  | Ciprofloxacin | NA | 0 | 100 |  | 0 | 8.8 | 91.2 |
|  | Colistin | 97.1 | NA | 2.9 |  | NA | NA | NA |
|  | Meropenem | 0 | 11.8 | 88.2 |  | 0 | 8.8 | 91.2 |
|  |  |  |  |  |  |  |  |  |
| Carbapenemase-negative (1,497)^c^ | Cefiderocol | 98.6 | NA | 1.4 |  | 95.2 | 3.4 | 1.4 |
|  | Aztreonam-avibactam (*n*=506)^d^ | NA | NA | NA |  | NA | NA | NA |
|  | Cefepime | NA | 56.3 | 43.7 |  | 56.3 | NA | 43.7 |
|  | Ceftazidime-avibactam | 86.1 | NA | 13.9 |  | 86.1 | NA | 13.9 |
|  | Ceftolozane-tazobactam | 89.0 | NA | 11.0 |  | 89.0 | 3.1 | 7.9 |
|  | Ciprofloxacin | NA | 36.3 | 63.7 |  | 36.3 | 10.6 | 53.1 |
|  | Colistin | 99.1 | NA | 0.9 |  | NA | NA | NA |
|  | Meropenem | 0 | 60.9 | 39.1 |  | 0 | 29.3 | 70.7 |

^a^Isolates may also harbor serine carbapenemases, ESBLs, or AmpC-type enzymes.

^b^Isolates may also harbor ESBLs or AmpC-type enzymes but not MBLs.

^c^Isolates may harbor ESBLs or AmpC-type enzymes but not carbapenemases.

^d^Aztreonam-avibactam was only tested in 2018 and 2019.

^e^NA, MIC breakpoints are not available.

**Table S4.** EUCAST and FDA susceptibility-testing interpretation for molecularly characterized, meropenem-nonsusceptible *Acinetobacter baumannii* complex isolates from SIDERO-WT surveillance study collected in North America and Europe from 2014 to 2019

| Genotype (no. of isolates) | Antimicrobial agent | EUCAST MIC interpretation | | |  | | FDA MIC interpretation | | | |  |
| --- | --- | --- | --- | --- | --- | --- | --- | --- | --- | --- | --- |
|  |  | % S | % I | % R | |  | | % S | % I | % R | |
| MBL (25)^a^ | Cefiderocol | NA^h^ | NA | NA | |  | | 24.0 | 20.0 | 56.0 | |
|  | Ampicillin-sulbactam (*n*=18)^e^ | NA | NA | NA | |  | | NA | NA | NA | |
|  | Cefepime (*n*=7)^f^ | NA | NA | NA | |  | | 0 | 0 | 100 | |
|  | Ceftazidime-avibactam | NA | NA | NA | |  | | NA | NA | NA | |
|  | Ceftolozane-tazobactam (*n*=7)^g^ | NA | NA | NA | |  | | NA | NA | NA | |
|  | Ciprofloxacin | NA | 28.0 | 72.0 | |  | | NA | NA | NA | |
|  | Colistin | 96.0 | 0 | 4.0 | |  | | NA | NA | NA | |
|  | Meropenem | 0 | 0 | 100 | |  | | 0 | 0 | 100 | |
|  |  |  |  |  | |  | |  |  |  | |
| OXA-23 group (1,783)^b^ | Cefiderocol | NA | NA | NA | |  | | 88.1 | 5.2 | 6.8 | |
|  | Ampicillin-sulbactam (*n*=333)^e^ | NA | NA | NA | |  | | NA | NA | NA | |
|  | Cefepime (*n*=1,449)^f^ | NA | NA | NA | |  | | 1.7 | 9.0 | 89.3 | |
|  | Ceftazidime-avibactam | NA | NA | NA | |  | | NA | NA | NA | |
|  | Ceftolozane-tazobactam (*n*=1,449)^g^ | NA | NA | NA | |  | | NA | NA | NA | |
|  | Ciprofloxacin | NA | 0.1 | 99.9 | |  | | NA | NA | NA | |
|  | Colistin | 81.4 | 0 | 18.6 | |  | | NA | NA | NA | |
|  | Meropenem | 0 | 0.7 | 99.3 | |  | | 0 | 0.2 | 99.8 | |
|  |  |  |  |  | |  | |  |  |  | |
| OXA-24 group (570)^b^ | Cefiderocol | NA | NA | NA | |  | | 75.1 | 8.9 | 16.0 | |
|  | Ampicillin-sulbactam (*n*=85)^e^ | NA | NA | NA | |  | | NA | NA | NA | |
|  | Cefepime (*n*=485)^f^ | NA | NA | NA | |  | | 8.2 | 24.5 | 67.2 | |
|  | Ceftazidime-avibactam | NA | NA | NA | |  | | NA | NA | NA | |
|  | Ceftolozane-tazobactam (*n*=485)^g^ | NA | NA | NA | |  | | NA | NA | NA | |
|  | Ciprofloxacin | NA | 0.7 | 99.3 | |  | | NA | NA | NA | |
|  | Colistin | 98.4 | 0 | 1.6 | |  | | NA | NA | NA | |
|  | Meropenem | 0 | 1.2 | 98.8 | |  | | 0 | 0.4 | 99.6 | |
|  |  |  |  |  | |  | |  |  |  | |
| OXA-58 group (69)^b^ | Cefiderocol | NA | NA | NA | |  | | 91.3 | 7.2 | 1.4 | |
|  | Ampicillin-sulbactam (*n*=9)^e^ | NA | NA | NA | |  | | NA | NA | NA | |
|  | Cefepime (*n*=60)^f^ | NA | NA | NA | |  | | 16.7 | 28.3 | 55.0 | |
|  | Ceftazidime-avibactam | NA | NA | NA | |  | | NA | NA | NA | |
|  | Ceftolozane-tazobactam (*n*=60)^g^ | NA | NA | NA | |  | | NA | NA | NA | |
|  | Ciprofloxacin | NA | 0 | 100 | |  | | NA | NA | NA | |
|  | Colistin | 85.5 | 0 | 14.5 | |  | | NA | NA | NA | |
|  | Meropenem | 0 | 72.5 | 27.5 | |  | | 0 | 4.3 | 95.7 | |
|  |  |  |  |  | |  | |  |  |  | |
| OXA-23 and 24 group (19)^b^ | Cefiderocol | NA | NA | NA | |  | | 89.5 | 10.5 | 0 | |
|  | Ampicillin-sulbactam (*n*=18)^e^ | NA | NA | NA | |  | | NA | NA | NA | |
|  | Cefepime (*n*=1)^f^ | NA | NA | NA | |  | | 0 | 0 | 100 | |
|  | Ceftazidime-avibactam | NA | NA | NA | |  | | NA | NA | NA | |
|  | Ceftolozane-tazobactam (*n*=1)^g^ | NA | NA | NA | |  | | NA | NA | NA | |
|  | Ciprofloxacin | NA | 0 | 100 | |  | | NA | NA | NA | |
|  | Colistin | 89.5 | 0 | 10.5 | |  | | NA | NA | NA | |
|  | Meropenem | 0 | 0 | 100 | |  | | 0 | 0 | 100 | |
|  |  |  |  |  | |  | |  |  |  | |
| OXA-23 and 58 group (34)^b^ | Cefiderocol | NA | NA | NA | |  | | 100 | 0 | 0 | |
|  | Ampicillin-sulbactam (*n*=13)^e^ | NA | NA | NA | |  | | NA | NA | NA | |
|  | Cefepime (*n*=21)^f^ | NA | NA | NA | |  | | 0 | 9.5 | 90.5 | |
|  | Ceftazidime-avibactam | NA | NA | NA | |  | | NA | NA | NA | |
|  | Ceftolozane-tazobactam (*n*=21)^g^ | NA | NA | NA | |  | | NA | NA | NA | |
|  | Ciprofloxacin | NA | 2.9 | 97.1 | |  | | NA | NA | NA | |
|  | Colistin | 97.1 | 0 | 2.9 | |  | | NA | NA | NA | |
|  | Meropenem | 0 | 2.9 | 97.1 | |  | | 0 | 2.9 | 97.1 | |
|  |  |  |  |  | |  | |  |  |  | |
| PER/VEB (103)^c^ | Cefiderocol | NA | NA | NA | |  | | 5.8 | 1.9 | 92.2 | |
|  | Ampicillin-sulbactam (*n*=10)^e^ | NA | NA | NA | |  | | NA | NA | NA | |
|  | Cefepime (*n*=93)^f^ | NA | NA | NA | |  | | 1.1 | 0 | 98.9 | |
|  | Ceftazidime-avibactam | NA | NA | NA | |  | | NA | NA | NA | |
|  | Ceftolozane-tazobactam (*n*=93)^g^ | NA | NA | NA | |  | | NA | NA | NA | |
|  | Ciprofloxacin | NA | 0 | 100 | |  | | NA | NA | NA | |
|  | Colistin | 100 | 0 | 0 | |  | | NA | NA | NA | |
|  | Meropenem | 0 | 4.9 | 95.1 | |  | | 0 | 2.9 | 97.1 | |
|  |  |  |  |  | |  | |  |  |  | |
| Carbapenemase-negative (309)^d^ | Cefiderocol | NA | NA | NA | |  | | 86.1 | 6.1 | 7.8 | |
|  | Ampicillin-sulbactam (*n*=62)^e^ | NA | NA | NA | |  | | NA | NA | NA | |
|  | Cefepime (*n*=247)^f^ | NA | NA | NA | |  | | 17.4 | 35.6 | 47.0 | |
|  | Ceftazidime-avibactam | NA | NA | NA | |  | | NA | NA | NA | |
|  | Ceftolozane-tazobactam (*n*=247)^g^ | NA | NA | NA | |  | | NA | NA | NA | |
|  | Ciprofloxacin | NA | 8.1 | 91.9 | |  | | NA | NA | NA | |
|  | Colistin | 98.1 | 0 | 1.9 | |  | | NA | NA | NA | |
|  | Meropenem | 0 | 40.8 | 59.2 | |  | | 0 | 19.4 | 80.6 | |

^a^Isolates may also harbor serine carbapenemases, ESBLs, or AmpC-type enzymes.

^b^Isolates may also harbor ESBLs or AmpC-type enzymes but not MBL or other OXA carbapenemases than those noted.

^c^Isolates may harbor OXA carbapenemases, ESBLs, or AmpC-type enzymes but not MBLs.

^d^Isolates may harbor ESBLs or AmpC-type enzymes but not carbapenemases.

^e^Ampicillin-sulbactam was only tested in 2019.

^f^Cefepime MIC data not available for 2019

^g^Ceftolozane-tazobactam was not tested in 2019.

^h^NA, MIC breakpoints are not available.
